# Service Users’ Views on Digital Remote Monitoring for Psychosis: Survey Study

**DOI:** 10.2196/86152

**Published:** 2026-05-05

**Authors:** Xiaolong Zhang, Emily Eisner, Daniela Di Basilio, Cara Richardson, Joseph Firth, Sandra Bucci

**Affiliations:** 1 Division of Psychology and Mental Health, School of Health Sciences, Faculty of Biology, Medicine and Health, Manchester Academic Health Science Centre, The University of Manchester, 1st Floor, Jean McFarlane Building, 176 Oxford Rd, Manchester, M13 9PY, United Kingdom, 44 1613060422; 2 Greater Manchester Mental Health NHS Foundation Trust, Manchester, United Kingdom; 3 Division of Health Research, Faculty of Health and Medicine, Lancaster University, Lancaster, United Kingdom

**Keywords:** digital remote monitoring, psychosis, service user, passive sensing, smartphone, wearable

## Abstract

**Background:**

Digital remote monitoring using smartphones and wearable devices is a promising solution for psychosis management, where precise, time-sensitive intervention is crucial. Combining active symptom monitoring (ASM) and passive sensing (PS) can support self-management by allowing remote, low-burden mental health monitoring.

**Objective:**

This study aimed to explore (1) views on collecting data using ASM and PS methods and comfort levels with different types of data gathered via these methods, (2) views on using smartphones and wearable devices in the context of mental health care, and (3) the ownership and usage of smartphones and wearable devices.

**Methods:**

We conducted a cross-sectional survey study with service users with psychosis in the United Kingdom between March 2023 and March 2024.

**Results:**

A total of 309 participants completed the survey. They reported mixed views on using ASM and PS technologies for monitoring mental health, with more participants endorsing the concept than opposing it (ASM: n=145, 46.9% and PS: n=132, 42.7%). However, the type of data gathered using these methods was an important factor. Collecting personal information was deemed less acceptable (*P*<.001) than other data types (physical health, mental health, environment, and nonpersonal device information).

**Conclusions:**

We found that participants were comfortable with using apps and wearables for digital remote monitoring, though personal information was less acceptable than other data types due to privacy and surveillance concerns. This highlights the importance of further exploring trust issues related to digital monitoring and ensuring that end users have choices regarding the types of data that digital systems gather and share with mental health services.

## Introduction

### Background

Conventional methods of monitoring and treating psychosis rely on service users recalling symptoms over the preceding period, often weeks or months, at a scheduled appointment time with a member of their care team [1,2]. However, the accuracy of symptom recall is often compromised due to infrequent clinic appointments in overstretched mental health services [3,4]. This poses challenges for psychosis management, where precise, time-sensitive treatment is crucial. In this context, mental health services are increasingly advocating for the adoption of digital tools to overcome the limitations of conventional assessment and intervention approaches [5].

Digital remote monitoring (DRM) in the context of mental health care refers to the use of digital technology to track and assess mental health symptoms remotely. It includes active symptom monitoring ([ASM]; self-reported data) and passive sensing ([PS]; sensor-derived data), enabling continuous, low-burden monitoring and the opportunity for timely, personalized interventions [3,6,7]. PS using smartphones and wearables can collect extensive sensor data, from which features can be extracted to infer clinical states [8]. In psychosis specifically, a recent systematic review found that the most used devices for PS were accelerometers and smartphones, and the most commonly derived features were sleep duration and time spent sedentary [9]. Despite being in its early stages, recent advancements in DRM for psychosis show its potential for assessment and treatment [10]. Specifically, it has been used to monitor psychotic symptoms [11,12], functional outcomes [12], and sleep [13]. Beyond symptom monitoring, digital tools have also been used for relapse prediction. A recent systematic review found that, as of 2022, 52 studies had used ASM or PS to track early warning signs and other antecedent behaviors of relapse or symptom worsening, reflecting the rapid growth of these technologies in mental health care [14].

Numerous surveys have demonstrated a continuous increase in digital technology ownership among individuals with psychosis [15-17]. A recent survey study reported a 90% smartphone ownership rate [18], suggesting that access to devices may no longer be a significant barrier to using digital health technologies in psychosis care. Moreover, the survey indicated that most participants used instant messaging and social media multiple times per day, while health-related apps were rarely used [18]. However, despite this accessibility, service user acceptance and engagement with digital health systems have remained low, partly due to the insufficient understanding of stakeholders’ preferences and needs [19]. Indeed, a recent review exploring the barriers and facilitators of engagement with digital mental health interventions (DMHIs) highlighted that successful implementation depends on the functionality of digital health systems, the relevance of their content to users’ specific needs, and users perceiving value and benefit from using these interventions [20]. Additionally, demographic and other factors may affect engagement, but their effects are inconsistent across studies [20]; for example, some studies found higher engagement among older adults [21,22], while others found higher engagement among younger people [23,24] or mixed effects [25]. While previous research has explored general barriers and facilitators to engagement, there is a gap in understanding perceptions of PS technologies in this population.

### Aims

This study aimed to understand the views of service users with psychosis on using personal digital devices for remote symptom monitoring. We aimed to explore (1) views on collecting data using ASM and PS methods specifically in the context of an individual’s mental health care, and comfort levels with different types of data gathered via these methods; (2) broad views on using smartphones and wearable devices in the context of mental health care; and (3) how individuals with psychosis own and use smartphones and wearable devices, as well as the barriers they perceive to their use. In this study, digital health tools refer to apps, sensing features on a smartphone, wearable devices, or smartwatches that are used for monitoring and managing mental and physical health (eg, monitoring symptoms, counting steps, helping with sleep routines, and setting medication reminders).

## Methods

### Participants

We conducted a one-off survey study with service users with psychosis between March 2023 and March 2024. To enhance inclusivity, we deliberately applied a broad criterion for psychosis, incorporating both the use of antipsychotic medication and the presence of psychotic symptoms (ie, hallucinations, delusions, confused, and disturbed thoughts) in addition to a formal diagnosis where available. More specifically, eligibility criteria were (1) self-reported diagnosis of a schizophrenia-spectrum disorder, taking antipsychotic medication, or experiencing psychotic symptoms; (2) aged 18 years or older; and (3) provision of informed consent. Participants not sufficiently fluent in English to complete the survey were excluded from participation. To reach a broad sample, we used a wide range of recruitment methods: social media platforms (eg, X (X Corp) and Reddit (Reddit, Inc), displaying posters in mental health services, asking clinicians to identify potential participants from their caseload, recruitment via the Clinical Research Network across England, existing research cohorts of the research team, and third sector organizations.

### Data Collection

The survey (Table S1 in Multimedia Appendix 1) took approximately 20‐30 minutes to complete and gathered demographic information and explored participants’ experiences with and opinions of using smartphone apps and wearable devices for mental health management. It also explored views on using digital health tools in clinical practice. The survey was developed based on a review of existing literature on DMHIs in mental health care [26-37]. To enhance participants’ understanding of DRM, the survey included brief explanations of ASM and PS, accompanied by images of various digital devices. Participants could choose to complete the questionnaire in either an online or paper format based on their preference. The Qualtrics (Qualtrics, LLC) platform [38] was used to deliver the online version, accessed via a URL link or a QR code. The participant information sheet was embedded in the survey for potential participants to read, followed by a series of screening questions to assess eligibility. After participants completed the screening questions, those who were not eligible were shown a thank-you message explaining their ineligibility, and the survey ended. Eligible participants were directed to the survey questions. For participants completing a paper questionnaire, a study pack containing the paper survey (identical in content to the online version) and a return postage-paid envelope was sent. Paper questionnaire responses were then entered manually into the Qualtrics database. To monitor data integrity and minimize the risk of survey fraud, study data were reviewed monthly by the research team. Summary completion numbers were shared with participating NHS trusts, and local contacts were asked to confirm that the number of surveys received aligned with their recruitment logs. Patient and public contributors in our digital mental health group reviewed survey items for acceptability and relevance.

### Data Analysis

Quantitative analysis was performed using R (R Core Team and R Foundation for Statistical Computing) [39]. Descriptive statistics, including frequencies and percentages, were calculated. To understand the differences in participants’ comfort level with different features collected via DRM, we used principal component analysis (PCA) to cluster features into high-level data-type groups (eg, personal data and health data). The clustering process was informed by a previous study on individuals’ perspectives on sharing different types of digital remote monitoring data [40]. Using this as a conceptual framework, an initial grouping of features was proposed and subsequently refined through iterative discussions among authors XZ, EE, and SB. PCA biplots were examined to assess how features grouped across components, and the pair of components that provided the clearest visual separation between feature groups was used to guide clustering. Following that, we conducted the Kruskal-Wallis test to compare participants’ comfort levels between data-type groups. Dunn post hoc test with Bonferroni correction was applied to identify the specific significant differences. *P*<.05 was considered statistically significant. Data visualization was created using R and Microsoft Excel [41]. We used content analysis to summarize the free-text data [42]. Initial coding was conducted by author XZ, and the results were subsequently reviewed and refined by authors EE and SB. When divergence occurred, a meeting was scheduled to discuss the discrepancies until agreement was reached. NVivo (version 12; QSR International Pty Ltd, now known as Lumivero) was used to analyze free-text data [43].

### Ethical Considerations

The study was approved by the North West–Greater Manchester West Research Ethics Committee (reference: 22/NW/0246). All participants had the option to enter a £50 (US $67.40) prize draw at the end of the survey as compensation for their time. A participant information sheet was embedded in the survey for potential participants to read.

## Results

### Demographics

In total, 309 participants completed the survey. The median age was 38.5 (IQR 28‐50) years. There was an approximately even gender split (male: n=145, 47% and female: n=153, 49%). Almost all participants reported their gender was the same as their sex assigned at birth (n=298, 96.4%). Most participants were White (n=244, 79%), single (n=173, 56%), and had no caring responsibilities (n=213, 68.9%). A third of participants were employed (n=99, 32%), over half were living with family (n=159, 51.5%), and nearly three-quarters had received further education or higher (n=223, 72.2%). Full sample demographic characteristics are shown in Table 1.

**Table 1.** Baseline characteristics and service use (N=309).

Characteristic	Value
Age (years), median (IQR)	38.5 (28-50)
Sex, n (%)	
Female	153 (49.51)
Male	145 (46.93)
Nonbinary or third gender	4 (1.29)
Other	3 (0.97)
Prefer not to say or unsure	1 (0.32)
Missing	3 (0.97)
Ethnicity, n (%)	
Asian or Asian British	22 (7.11)
Black, Black British, Caribbean, or African	18 (5.82)
White	244 (78.96)
Other ethnic group	3 (0.97)
Mixed or multiple ethnic groups	15 (4.85)
Prefer not to say	1 (0.32)
Missing	6 (1.94)
Relationship, n (%)	
Single	173 (55.99)
Cohabiting	14 (4.53)
In a relationship	41 (13.27)
Married	48 (15.53)
Divorced	11 (3.56)
Widowed	3 (0.97)
Other	1 (0.32)
Prefer not to say	10 (3.24)
Missing	8 (2.59)
Job, n (%)	
Employed	99 (32.04)
Full-time parent or carer	3 (0.97)
Homemaker	2 (0.65)
Out of work and looking for work	16 (5.18)
Out of work and not looking for work	16 (5.18)
Retired	17 (5.50)
Student	22 (7.12)
Unable to work	43 (13.92)
Unemployed	61 (19.74)
Voluntary work	15 (4.85)
Prefer not to say	3 (0.97)
Other	4 (1.29)
Missing	8 (2.59)
Education, n (%)	
Primary school	6 (1.94)
Secondary school (up to GCSEs^a^)	59 (19.09)
Further education (sixth form, college, or equivalent)	78 (25.24)
Trade, technical, or vocational training	11 (3.56)
Diploma or equivalent	38 (12.30)
University bachelor’s degree	52 (16.83)
University master’s degree	31 (10.03)
PhD or higher	13 (4.21)
Other	3 (0.97)
Prefer not to say	10 (3.24)
Missing	8 (2.59)
Living arrangements, n (%)	
Living alone	106 (34.30)
Living with family	159 (51.46)
Living with others (eg, friends)	11 (3.56)
Living in supported housing	13 (4.21)
Other	10 (3.24)
Prefer not to say	3 (0.97)
Missing	7 (2.27)
Care duties, n (%)	
No	213 (68.93)
Yes	77 (24.92)
Missing	19 (6.15)

a GCSE: General Certificate of Secondary Education.

### Views on Collecting Data Using Active Monitoring and PS Methods

As shown in Table 2, a greater number of participants endorsed using ASM and PS for remote monitoring (ASM: n=145, 46.9% and PS: n=132, 42.7%) and sharing this information with their care team (ASM: n=144, 46.6% and PS: n=134, 43.4%) compared to those who opposed it. Nevertheless, many participants felt uncomfortable using these technologies for data collection (ASM: n=56, 18.1% and PS: n=73, 23.6%) and sharing the data with their care team (ASM: n=61, 19.7% and PS: n=60, 19.4%), and a notable number of participants found these technologies unhelpful (ASM: n=45, 14.6% and PS: n=47, 15.2%). For the questions reported in Table 2, a substantial proportion of participants reported “unsure” (range 18.5%‐21.7%) or did not answer the questions (range 14.6%‐15.5%).

**Table 2.** Comfort with and perceived helpfulness of digital monitoring via active symptom monitoring and passive sensing.

Items	Active symptom monitoring, n (%)	Passive sensing, n (%)
How comfortable do you feel with university researchers using it to collect information about your feelings, behaviors, general health, or whereabouts? (please tick to indicate)		
Comfortable	145 (46.93)	132 (42.72)
Uncomfortable	56 (18.12)	73 (23.62)
Unsure	63 (20.39)	57 (18.45)
Missing	45 (14.56)	47 (15.21)
How helpful would it be in helping you manage your mental health or well-being?		
Helpful	128 (41.42)	133 (43.04)
Unhelpful	45 (14.56)	47 (15.21)
Unsure	91 (29.45)	82 (26.54)
Missing	45 (14.56)	47 (15.21)
Would you be comfortable with information about whether you have been sleeping, exercising, traveling, or phoning people being collected via…		
Smartphone	37 (11.97)	37 (11.97)
Wearable device	34 (11)	25 (8.09)
Both	126 (40.78)	115 (37.22)
Neither	67 (21.68)	85 (27.51)
Missing	45 (14.56)	47 (15.21)
How comfortable do you feel with information about whether you have been sleeping, exercising, traveling, or phoning people being collected and shared with a member of your clinical or mental health team?		
Comfortable	144 (46.6)	134 (43.37)
Uncomfortable	61 (19.74)	60 (19.42)
Unsure	59 (19.09)	67 (21.68)
Missing	45 (14.56)	48 (15.53)

**Figure 1.**
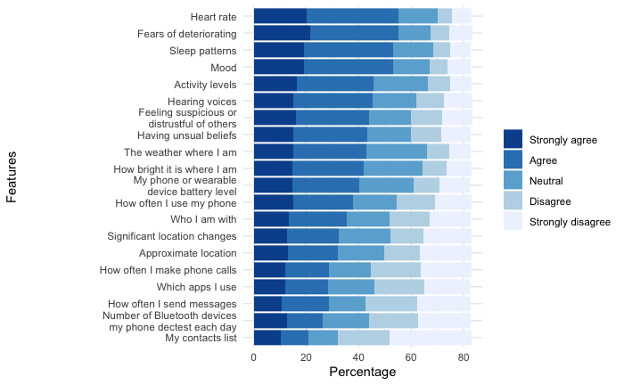
Acceptability of collecting specific types of information using a smartphone or wearable device.

Figure 1 shows participants’ responses regarding the acceptability of using a smartphone or wearable to collect 20 specific types of information. Only 4 out of the 20 data types or features received over 50% endorsement by participants. Specifically, heart rate (55%), fears of deteriorating (55%), sleep patterns (53%), and mood (53%) were endorsed (“agree” or “strongly agree”) by participants. PCA was performed to further explore participants’ comfort levels with different categories of data. Although principal components 1 and 2 explained most of the variability (66.6% and 11.1%, respectively), the clusters on the biplot of these 2 components were not clear (refer to Figure S1 in Multimedia Appendix 1). Instead, we used the biplot of principal component 2 and principal component 3 (which explained 4.3% of the variability) to guide the clustering (refer to Figure S2 in Multimedia Appendix 1). Features were clustered into 5 data types: physical health information (eg, heart rate), mental health information (eg, mood), personal information (eg, location), environmental information (eg, weather), and nonpersonal device information (eg, battery level). The Kruskal-Wallis test showed that participants’ comfort levels differed significantly between data-type groups (*P*<.001). Dunn post hoc test for pairwise comparisons showed that participants were significantly less comfortable sharing personal information compared to all other data types (*P*<.001). Participants felt significantly more comfortable sharing mental health and physical health data than for all other data types (*P*<.05), except for environmental data (no significant difference between mental health data and environmental data; *P*≥.99). There was no significant difference in comfort levels between sharing mental health data and physical health data (*P*=.24), nor between environmental data and nonpersonal device data (*P*=.57; refer to Table 3).

**Table 3.** Pairwise comparison of participants’ comfort level with sharing different types of data with researchers.

Comparison	*z* value	Unadjusted *P* value	Adjusted *P* value^a^
Environmental information–nonpersonal device information	1.91	.06	.57
Environmental information–mental health information	−1.17	.24	≥.99
Nonpersonal device information–mental health information	−3.45	.001	.006
Environmental information–personal information	8.08	<.001	<.001
Nonpersonal device information–personal information	5.67	<.001	<.001
Mental health information–personal information	12.94	<.001	<.001
Environmental information–physical health information	−2.87	.004	.04
Nonpersonal device information–physical health information	−4.96	<.001	<.001
Mental health information–physical health information	−2.25	.02	.24
Personal information–physical health information	−13.31	<.001	<.001

a *P* values were adjusted for multiple comparisons using the Bonferroni correction.

Participants were also asked to share any other concerns not listed in the survey in a free-text question (refer to Tables S2 and S3 in Multimedia Appendix 1). The top concerns about ASM were “risk of data misuse” (29/91 free-text responses, 32%), “feeling uncomfortable with being monitored” (19/91, 21%), and “tracking symptoms making people feel worse” (14/91, 15%). For PS, participants expressed concerns such as “feeling uncomfortable with passive sensing” (19/57 free-text responses, 33%), “it is invasive and unhelpful” (13/57, 23%), and “preferring to use it collaboratively rather than being monitored by a clinician” (9/57, 16%).

### Views on Using Smartphones and Wearable Devices in the Context of Mental Health Care

The concept of using an app or a wearable device to support mental health between appointments was highly acceptable. A notable 59.9% (n=185) were willing to use an app, while an even higher 65% (n=201) expressed interest in using a wearable device. When it comes to integrating apps or wearables into mental health management, the most favored option was a blended approach of either an app (n=124, 51.7%) or a wearable (n=102, 41.1%) combined with face-to-face care. Moreover, the preferred method for receiving prompts to complete symptom monitoring tasks was a combination of prescheduled prompts and self-initiated responses, rather than relying solely on either method. Participants preferred this approach for both smartphone apps (n=86, 35.8%) and wearable devices (n=74, 29.8%).

Regarding the perceived impact of using apps and wearables, more than half of the participants reported that using such technologies would have no negative impact on their mood (apps: n=135, 56.3% and wearables: n=140, 56.5%) or psychotic symptoms (apps: n=117, 48.8% and wearables: n=144, 58.1%). However, when asked about the positive impact, half of the participants expressed a neutral stance on the statement that using a smartphone app (n=121, 50.4%) or a wearable device (n=128, 51.6%) makes them feel happy. Over half (n=130, 54.2%) reported that using smartphone apps could help them feel connected with others, whereas slightly fewer (n=122, 49.2%) participants reported this for wearable devices. In terms of impacts on overall mental health and well-being, 40.8% (n=98) and 37.9% (n=94) either “agreed” or “strongly agreed” that using smartphone apps and wearable devices, respectively, could help their overall mental health and well-being. A small proportion of participants considered using smartphone apps (n=48, 20%) or wearable devices (n=50, 20.2%) to be unhelpful for overall mental health and well-being.

As shown in Table S4 in Multimedia Appendix 1, the ranking of advantages, based on the total percentage of participants who reported “agree” or “strongly agree,” was consistent across both smartphones and wearables. Top advantages were the ability to be used at any time, in any location (app: 189/240, 78.8% participants and wearables: 193/248, 77.8% participants), sharing information in real time with the care team (app: 180/240, 75% participants and wearables: 180/248, 72.6% participants), the ability to identify triggers and patterns (app: 171/240, 71.3% participants and wearables: 172/248, 69.4% participants), and being able to record and reflect on symptoms and experiences over time (app: 176/240, 73.3% participants and wearables: 168/248, 67.7% participants). Additional advantages specific to smartphone apps are shown in Table S5 in Multimedia Appendix 1. When participants were asked the extent to which barriers might impact their likelihood of using smartphone apps or wearable devices for mental health care (refer to Table S6 in Multimedia Appendix 1), the primary concern for apps was that they might be used as an excuse to replace face-to-face care (141/240, 58.8% participants). In contrast, cost was the main barrier for wearables (144/248, 58.1% participants).

### Ownership and Usage of Smartphones and Wearable Devices

Of the 309 participants, 276 (89.3%) owned or had access to a smartphone; in contrast, 95 (30.7%) participants owned or had access to a wearable device. The percentages of iPhone (n=138, 44.7%) and Android (Google LLC; n=141, 45.6%) smartphone users were comparable. For participants using a wearable device, Apple Watch was the most used device (n=32, 10.4%), followed by Fitbit (n=28, 9.1%) and Samsung (n=16, 5.2%). Most participants reported that they faced no barriers to owning or using a smartphone (n=191, 61.8%). For those who did encounter barriers, paranoia or suspiciousness related to technology (n=37, 12%), affordability (n=32, 10.4%), and digital literacy (n=27, 8.7%) were the top reasons. Most participants (n=215, 69.6%) identified barriers to owning or using a wearable device. The primary barrier was a lack of interest (n=94, 30.4%), followed by affordability (n=82, 26.5%) and a perceived lack of need to use it (n=45, 14.6%). In terms of frequency of use, smartphones and smartphone apps were the most frequently used digital tools, with 77% (n=238) and 60.2% (n=186) of participants, respectively, reporting using these multiple times a day. In contrast, in line with the ownership rates, 60.5% (n=187) stated they did not use wearable devices. Nearly half of the participants (n=147, 47.6%) had used health-related apps. Among those, physical health apps were the most used (n=99, 32%), while just over a quarter (n=80, 25.9%) reported using mental health apps.

Regarding participants’ preferences for the appearance of wearable devices, we selected Fitbit as an example of a consumer-grade device and the Empatica E4 as an example of a research-grade device. These 2 devices were chosen because they are frequently used in digital mental health research [9]. More participants chose the Fitbit (n=168, 54.4% participants) with a black strap (n=106, 50.7% participants) due to its more stylish design compared to the Empatica E4 (n=41, 13.3% participants), Fitbit with steel blue strap (n=52, 24.9% participants), and Fitbit with lunar white strap (n=49, 23.4% participants). Regardless of which type of device they selected, most participants were willing to use it daily (E4: 25/41, 61% participants and Fitbit: 121/168, 72% participants) and for longer than 1 year (E4: 21/41, 51.2% participants and Fitbit: 87/168, 51.8% participants). Participants identified the most important features of wearable devices as privacy (n=186, 89% participants), physical comfort (n=185, 88.5% participants), reasonable price (n=181, 86.6% participants), long battery life (n=181, 86.6% participants), and ease of use (n=174, 83.3% participants). A full list of features, ranked by importance, is shown in Table S7 in Multimedia Appendix 1.

## Discussion

### Principal Findings

We conducted a one-off survey with a large sample of individuals in the United Kingdom who self-reported a diagnosis of schizophrenia-spectrum disorder, were taking antipsychotic medication, or were experiencing psychotic symptoms, to gather their perspectives on DRM of symptoms, including ASM and PS, using smartphone apps and wearable devices. Both smartphone apps (59.9%) and wearable devices (65%) were considered acceptable tools for digital monitoring in mental health care. Most participants preferred a blended approach that combined digital tools with in-person support and were willing to use wearable devices daily and for longer than a year to manage their mental health. Nevertheless, although more participants accepted than rejected the use of ASM and PS technologies for monitoring than those who opposed it, the acceptance rate was still below 50%. This suggests that user-level barriers must be addressed to facilitate the adoption of these methods in mental health care. Consistent with previous studies [17,18,44], smartphone ownership rates were high, and smartphones were frequently used, with participants reporting few barriers to owning or using one. In contrast, wearables were less commonly owned, less frequently used, and participants faced more barriers (eg, cost) to owning or using them. To further investigate the persistent barriers to accessibility and acceptability of digital interventions, qualitative studies, or the incorporation of qualitative interviews alongside quantitative surveys in mixed methods research would be valuable for elucidating nuanced contextual factors that may influence uptake.

Our sample was predominantly White (79%), with fewer participants from ethnic minority groups (eg, Black, 5.8%), which is less representative of the ethnic diversity typically observed in populations experiencing psychosis [45]. Additionally, 32% of participants were employed, which is higher than the employment rate reported in the broader psychosis population, which ranges from 1% to 18.5% [46]. Although the gender distribution in our sample was relatively balanced, we recruited slightly more females than males (49.5% vs 46.9%), diverging from the general trend in psychosis incidence, which is typically higher among males [47]. These differences suggest the need for further research to better understand the views of underrepresented groups on the use of personal digital devices for remote symptom monitoring.

Participants expressed varying levels of comfort with sharing different types of data with a research team. The most acceptable data types to be collected from a DRM tool were “physical health data” and “mental health data,” such as heart rate, fears of deteriorating, sleep patterns, and mood. Conversely, the least acceptable types of data were “personal information,” including contact lists, frequency of sending messages, and app usage. This is in line with a previous survey study on the general population’s comfort levels with sharing PS data for assessing depression and anxiety [40]. The study, which examined preferences for data sharing with clinicians, electronic health records (EHRs), and family members, found that participants were significantly more comfortable sharing health-related information (physical activity, mood, and sleep) than personal information (communication logs, location, and social activity) across all 3 recipient groups [40]. One explanation is that people might be more comfortable sharing health-related data because they see it as clinically relevant, whereas passive monitoring of personal behaviors (eg, communication and location) may feel more intrusive and less clinically relevant. Additionally, free-text responses in our survey study revealed that some participants were concerned about data misuse, disliked being monitored, and felt that monitoring was invasive and unhelpful. Taken together, the findings also indicate that privacy concerns remain a significant issue for people and pose a barrier to implementing digital monitoring in clinical practice. Some researchers have argued that training and explanation of the purpose of tracking to end users may reduce concerns [48]. However, our findings suggest that building trust may be more difficult than simply explaining the nonharmful nature of data tracking. Additionally, the complexity of methods such as machine learning, which is used to analyze large volumes of data collected from digital tools, makes it challenging even for clinicians to fully understand how the data will be used [49]. As the field moves toward collecting large-scale data from diverse populations, building trust between service users and digital systems is crucial.

Regarding common concerns about the potential negative effects of digital technologies on mental health, over half of the participants reported that using apps and wearables did not worsen their mood or psychotic symptoms. More participants found these technologies helpful for mental health and well-being rather than unhelpful and found that apps and wearables fostered a sense of connection, though a substantial proportion did not endorse such statements. These findings are consistent with a previous survey study [18], highlighting the complex relationship between technology use and mental health. Furthermore, serious negative effects, such as adverse events, remain an area of uncertainty. A recent review noted that while adverse events are rarely recorded and reported in DMHI trials, it is unclear whether this is due to a lack of occurrence or inadequate reporting [50]. Therefore, continued research is needed to better understand and monitor potential risks, ensuring the safety of digital health technologies in different populations.

Many participants had not used any health-related apps (42.4%). This finding echoes a previous US survey, which found that service users with psychosis rarely used their digital devices for health-related purposes, such as mental health symptom monitoring (14%) and sleep tracking (14%) [51]. Additionally, we found that for participants who had used a health-related app, physical health apps were more commonly used than mental health apps. Similarly, previous studies have identified high levels of interest in using physical health apps among young people with psychosis [52,53]. Given the increased risk of physical health conditions in individuals with severe mental health problems [54], and the frequent neglect of these issues in current mental health services [54,55], integrating both mental and physical health features in the same app may be preferred by service users. Moreover, most participants preferred DRM tools to be blended with face-to-face care. This is consistent with recent research highlighting the growing emphasis on blended care models, where digital tools complement traditional in-person services to enhance accessibility, engagement, and continuity of mental health support [56-58].

Despite the high rates of device ownership, a substantial proportion of participants reported that they faced barriers to owning or using smartphones and wearables. Specifically, for smartphones, paranoia or suspiciousness related to technology, affordability, and digital literacy were the top reasons, whereas for wearables, top barriers were a lack of interest, affordability, and a perceived lack of need to use them. These findings suggest that wearables may be perceived as less essential for daily life compared to smartphones. However, affordability was reported as a barrier for both technologies, suggesting that cost limits access to digital technology for individuals with psychosis. A recent systematic review indicated that providing supportive infrastructure to users, including devices and free internet access, is a potential solution for advancing digital health equity [59]. For example, in the United Kingdom, the NHS could directly provide devices through its funding, as using personal health budgets for device provision is feasible in NHS City and Hackney [60]. Therefore, device provision in standard practice should be considered if digital mental health becomes an essential component of mental health care.

### Strengths and Limitations

We reached a large, geographically diverse sample, enhancing the robustness and generalizability of our findings. We explicitly asked individuals who experience psychosis about their perspectives on PS, a rapidly developing subfield of digital mental health that has been largely unexplored in previous research. Regarding limitations, we relied on participants to self-report their diagnosis or experience of psychosis without independent validation. Also, this self-selected sample may have been more familiar, interested, and comfortable with using digital tools than the wider clinical population, as individuals who choose to participate in research are often more engaged with services. Moreover, the missing data rate of some questions was substantial, ranging from 1.3% to 15.5%, which can affect the reliability and validity of the findings. Additionally, although the survey included some free-text items, most data were quantitative, collected through structured response formats. As such, the study may not capture the depth or nuance that qualitative methods typically provide when exploring service user views in detail. Finally, our sample lacked ethnic diversity, with most participants being White, and almost all participants were in contact with mental health services. The findings may not be generalizable to individuals from diverse backgrounds, who may have different experiences, preferences, and barriers related to digital health technologies. Future research should aim to include more diverse samples to ensure that DMHIs are equitable and accessible to all service users.

### Conclusions and Clinical Implications

Given that certain types of personal information (eg, contact lists, message frequency, and app usage) are perceived as less acceptable for collection, DRM should prioritize transparent data governance, user consent, and customizable privacy settings to build trust and alleviate concerns about surveillance and data misuse. Developers need to adhere to regulatory standards, such as the Digital Technology Assessment Criteria [61], throughout the process of designing and developing DRM tools. Additionally, service users should have control over the data they share, with whom, and for what purpose, for instance, through implementing digital dashboards to present key health metrics to service users and allow for their active participation in care management and decisions over data sharing with clinical teams. Alongside this, clinicians should also play a role in explaining the purpose of DRM (what data is being collected, why, and what it will be used for), emphasizing its clinical benefits and safety profile while addressing potential privacy-related apprehensions. Although most participants did not perceive DRM as harming mental health, a substantial proportion expressed concerns. Therefore, clinicians should proactively assess potential unintended effects, such as increased anxiety, paranoia, or distress related to feeling monitored, and implement strategies to ensure the safe and supportive use of these technologies. The higher reported usage of physical health apps compared to mental health apps highlights the potential for developing integrated platforms that address both mental and physical health needs. Given the high prevalence of physical health problems in psychosis, combining mental health tracking with physical health features (eg, activity tracking, sleep monitoring, and medication reminders) could enhance user engagement and support holistic care. To ensure the practical integration of such platforms into existing clinical workflows, it is essential to leverage established digital infrastructures, particularly EHRs. Integrating digital health apps with EHRs would allow clinicians to seamlessly access and interpret integrated data on both physical and mental health, supporting comprehensive and person-centered care. Adopting interoperability standards would further support data exchange across health care settings, enabling the holistic management of physical and mental health within routine practice. By incorporating these considerations, integrated platforms have the potential to significantly enhance service user outcomes by enabling the early detection of both psychiatric symptom deterioration and cardiometabolic risks, thereby facilitating more personalized and timely interventions. Blended approaches, using DRM alongside face-to-face care, should be considered as a priority for clinical implementation, as it appears to be participants’ preference. Finally, should digital technologies become important in improving clinical care, they should be provided at no cost to service users, especially wearables, as cost appears to be a main barrier to owning or using this technology.

## Supplementary material

10.2196/86152 Multimedia Appendix 1 Service user survey and additional analyses.

## Abbreviations

ASM: active symptom monitoring
DMHI: digital mental health intervention
DRM: digital remote monitoring
EHR: electronic health record
PCA: principal component analysis
PS: passive sensing
