# Supplementary material for: Service Users’ Views on Digital Remote Monitoring for Psychosis: Survey Study
Source: JMIR Hum Factors. 2026 May 5;13:e86152. doi: 10.2196/86152 (PMC13143164; doi:10.2196/86152)
Supplement: Multimedia Appendix 1 [file humanfactors-v13-e86152-s001.docx]

Table of Contents

[Table S1. Service users survey 2](#_Toc193186004)

[Figure S1. Principal component analysis of participant comfort with sharing data: PC 1 and PC 2 38](#_Toc193186013)

[Figure S2. Principal component analysis of participant comfort with sharing data: PC 2 and PC 3 39](#_Toc193186014)

[Table S2. Concerns about collecting data using active monitoring (total number of references = 91) 40](#_Toc193186015)

[Table S3. Concerns about collecting data using passive sensing methods (total number of references = 57) 41](#_Toc193186016)

[Table S4. Potential advantages of smartphone apps/wearable devices for mental healthcare: N (%) 42](#_Toc193186017)

[Table S5. Additional advantages specific to smartphone apps (n = 240): N (%) 44](#_Toc193186018)

[Table S6. Barriers to using smartphone apps/wearable devices for mental healthcare: N (%) 45](#_Toc193186019)

[Table S7. Perceived importance of certain features of wearable devices (n = 209): N (%) 47](#_Toc193186020)

### **Table S1. Service users survey.**

# Screening Questions

If you are still interested in participating after reading the study information, please complete the following questions.

| Are you aged 18 or over? | - Yes - No |
| --- | --- |
| Have you ever received any of the following diagnoses [select as many as apply]? | - Psychosis - Schizophrenia (or “paranoid schizophrenia”), - Schizoaffective disorder, - Schizophreniform, - Depression with psychotic features (depression with unusual experiences like hallucinations and delusions), - Delusional disorder, - Bipolar disorder with psychotic experiences, - Brief psychotic disorder or - Any other disorder which included psychotic experiences - Don’t know - No |
| Are you currently taking antipsychotic medication for your mental health? [e.g. hearing voices, unusual and distressing beliefs, feeling suspicious/distrustful of others] | - Yes - No |
| Have you ever received mental health support or treatment for any of the following [select as many as apply]? | - Hearing voices, visions (e.g. Hallucinations) - Unusual and distressing beliefs (e.g. Delusions) - Feeling suspicious/distrustful of others (e.g. Paranoia) - No |
| Thank you! Please note that by continuing to the survey, you are providing informed consent to participate in the study, having read and understood the information above.  If you decide to take part, you will then be taken to the survey questions. Your data will be kept on a secure University server, accessed only by the study research team. | |
| Thank you for your interest. Unfortunately, if you have answered no to any of the diagnosis or treatment questions, you are not eligible to take part in this study. Please do not continue the survey, as we will be unable to use those answers.  If you would like to know more about why you are not eligible for the study, please email a member of the research team: [Name: Xiaolong Zhang  Contact details: [xiaolong.zhang@postgrad.manchester.ac.uk](mailto:xiaolong.zhang@postgrad.manchester.ac.uk)] | |

# Part 1: Demographic Information

| 1. ***Please select your gender***   Do you identify as:   - Woman / Female - Man / Male - Non-binary/third gender - Prefer not to say/unsure - Other (please state):   *Does your gender match with your sex assigned at birth?*   - Yes - No - Prefer not to say | 1. ***How old are you?***   ……………………………………………………………. |
| --- | --- |
| 1. ***What is your ethnic origin?***   **White**   - English, Welsh, Scottish, Northern Irish or British - Irish - Gypsy or Irish Traveller - Roma - Any other White background   **Asian or Asian British**   - Indian - Pakistani - Bangladeshi - Chinese - Any other Asian background   **Black, Black British, Caribbean or African**   - Caribbean - African - Any other Black, Black British, or Caribbean background   **Mixed or multiple ethnic groups**   - White and Black Caribbean - White and Black African - White and Asian - Any other Mixed or multiple ethnic background   **Other ethnic group**   - Arab - Any other ethnic group - Prefer not to say | 1. ***Are you in a relationship?***  - Single - Co-habiting - In a relationship - Married - Civil partnership - Divorced - Widowed - Prefer not to say - Other. Please state………………. |
| 1. ***Do you have a job or work at the moment?***  - Employed - Self-employed - Out of work and looking for work - Out of work but not looking for work - Parent or carer - Homemaker - Voluntary work - Student - Retired - Unable to work - Other – please specify:  ……………………….. - Prefer not to say | 1. ***What is your highest completed level of education?***  - Primary school - Secondary school (up to GCSEs) - Further education (Sixth form, college or equivalent vocational education) - Diploma or equivalent - Trade/technical/vocational training - University bachelor’s degree - University master’s degree - PhD or higher - Other education (Please specify): ....................................... - Prefer not to say |
| 1. ***Who do you usually live with?***  - Living alone - Living with a partner - Living with children - Living with a partner and children - Living with parent(s)/carer(s) - Living with other relatives - Living with others (eg, friends) - Living in supported housing - Prefer not to say - Other – please state: ............................. | 1. ***Do you have any children or people you care for? (For example, are you a parent or carer?)***  - Yes - No - Would rather not say/unsure   If you do care for people, who do you care for?   - Child/ren - Parent/s - Other. Please state…………. - Would rather not say |

# Part 2: Technology Ownership/Use Questions

## What are Digital Health Tools?

In this survey, we are asking about apps, sensing features on a smartphone, wearable devices, or smartwatches that are used for things like monitoring symptoms, counting steps, helping with sleep routine, and setting medication reminders. We are interested in how these devices are used for things like their blood sugar levels, heart rhythm, or their location (whereabouts).

Here are some pictures of different digital health tools:


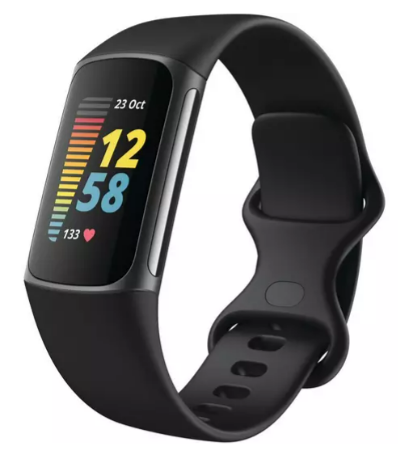

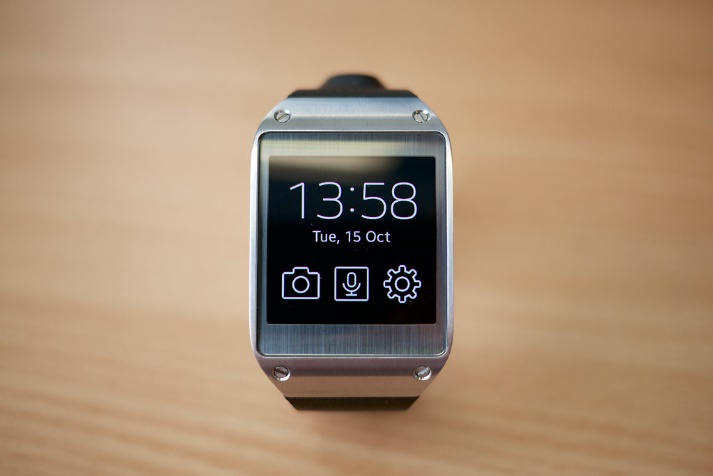


"Samsung Galaxy Gear smartwatch" by Janitors is marked with CC BY 2.0.

“Apple Watch" by wiyre.com is licensed under CC BY 2.0


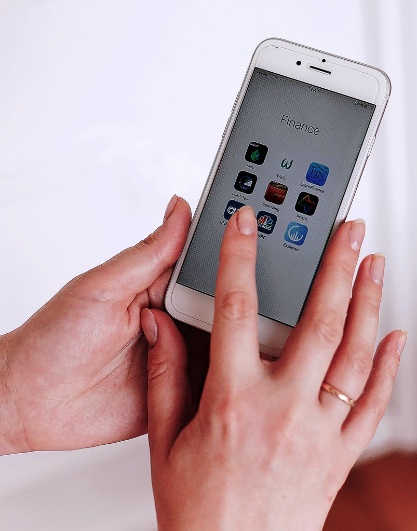


Smartphone apps" by Image Genie is marked with CC BY-SA 2.0.

1. Which of the following do you own or have access to? (Please tick all that apply)

- Smartphone – iPhone
- Smartphone – Android
- Smartphone – Other/ Don’t Know
- Tablet – iPad
- Tablet – Android
- Tablet – Other/Don’t Know
- None

1. On your phone, tablet, or wearable device (such as an Apple Watch or Fitbit), do you use any apps related to any of the things below?

(Please tick all that apply)

- - Mental health
  - Physical Health
  - Wellbeing
  - Mood and/or Health Tracking
  - Other health-related app
  - None

**If yes, which health-related app/apps do you use:**…………………………………..

1. Do you own or have access to any of the following wearable devices/smartwatches? (Please tick all that apply)

- Wearable device/smartwatch – Fitbit
- Wearable device/smartwatch – Apple
- Wearable device/smartwatch – Garmin
- Wearable device/smartwatch – Samsung
- Wearable device/smartwatch – Fossil
- Wearable device/smartwatch – other (Please state) …………………..
- None

1. How often do you use the following? (Please tick to indicate)

| Mobile phone (that’s not a smartphone) | Multiple times a day | Once a day | A few times a week | Once a week | A few times a month | Once a month | Less often | N/A I do not use this |
| --- | --- | --- | --- | --- | --- | --- | --- | --- |
| Smartphone | Multiple times a day | Once a day | A few times a week | Once a week | A few times a month | Once a month | Less often | N/A I do not use this |
| Smartphone apps (e.g. Facebook, Twitter, Instagram, Headspace) | Multiple times a day | Once a day | A few times a week | Once a week | A few times a month | Once a month | Less often | N/A I do not use this |
| Smartwatch/fitness tracker (e.g. Apple watch or Fitbit) | Multiple times a day | Once a day | A few times a week | Once a week | A few times a month | Once a month | Less often | N/A I do not use this |

1. What are the barriers you have faced, if any, to being able to own or use a wearable device? (Please tick all that apply):

- I struggle to afford to own and/or use a wearable device
- I’m not interested in wearable devices
- I don’t need to use a wearable device
- I keep losing or damaging wearable devices
- I don’t know how to use a wearable device
- I don’t know how to use certain wearable device features (eg, exercise tracking)
- I feel paranoid or suspicious about wearable devices
- Not applicable (there are no barriers for me)
- Other (please state) ………………………

1. What are the barriers you have faced, if any, to being able to own or use a smartphone? (Please tick all that apply):

- I struggle to afford to own and/or use a smartphone
- I’m not interested in smartphones
- I don’t need to use a smartphone
- I keep losing or damaging smartphones
- I don’t know how to use a smartphone
- I don’t know how to use certain smartphone features (eg, apps)
- I feel paranoid or suspicious about smartphones
- Not applicable (there are no barriers for me)
- Other (please state) ………………………

# Part 3: Using Digital Health Tools

1. If a member of your clinical/mental health team asked you to use a wearable device to help you manage your mental health between medical/health appointments (i.e. in your day-to-day life), would you wear/use it? (please tick to indicate)

Yes No (if no, please skip to Q24) Unsure

1. If you were offered a wearable device as part of your mental health care, is there any extra mental health support you would want to receive? (please tick to indicate)
   - I would be happy to use the wearable device on its own with no other mental health support
   - I would want to use the wearable device in conjunction with remote mental health support (eg, therapy/Dr appointments over the phone / video-calling)
   - I would want to use the wearable device in conjunction with face-to-face support/therapy/appointments
   - I would not want to use a wearable device as part of my mental health care
2. To what extent do you agree or disagree with the following statements about wearable devices for mental healthcare (1 = strongly disagree; 5 = strongly agree)?

| **Question** | **Strongly disagree** | **Disagree** | **Neutral** | **Agree** | **Strongly agree** |
| --- | --- | --- | --- | --- | --- |
| Using a wearable device makes me depressed or anxious | 1 | 2 | 3 | 4 | 5 |
| Using a wearable device makes me happy | 1 | 2 | 3 | 4 | 5 |
| Using a wearable device makes me feel paranoid or suspicious | 1 | 2 | 3 | 4 | 5 |
| Using a wearable device helps me to feel connected | 1 | 2 | 3 | 4 | 5 |
| Using a wearable device helps my overall mental health and wellbeing | 1 | 2 | 3 | 4 | 5 |
| Using a wearable device is unhelpful for my overall mental health and wellbeing | 1 | 2 | 3 | 4 | 5 |

1. If a member of your clinical/mental health team asked you to use/wear a wearable device (e.g. Fitbit or Apple watch) to help manage your mental health, to what extent do you agree or disagree with the following statements (1 = strongly disagree; 5 = strongly agree)?

| **Question** | **Strongly disagree** | **Disagree** | **Neutral** | **Agree** | **Strongly agree** |
| --- | --- | --- | --- | --- | --- |
| I would like a wearable device to alert/remind me about healthcare appointments | 1 | 2 | 3 | 4 | 5 |
| I would like a wearable device to alert/remind me to take my medication | 1 | 2 | 3 | 4 | 5 |
| I would a wearable device to prompt me to record how I am feeling | 1 | 2 | 3 | 4 | 5 |

1. Wearable devices can prompt/remind you to answer questions about your thoughts/feelings/behaviours/general health/whereabouts. Would you prefer to receive reminders to answer questions about your thoughts/feelings, etc from a wearable device, or would you prefer to answer these questions in your own time (ie, without being prompted/reminded)?

- I would like to receive prompts/reminders
- I would like to answer questions in my own time (ie, when I choose)
- I would like a combination
- I don’t mind
- Neither

1. To what extent do you agree or disagree (1 = strongly disagree; 5 = strongly agree) that the following are potential advantages of wearable devices for mental healthcare? (Please tick all that apply)

|  | **Strongly disagree** | **Disagree** | **Neutral** | **Agree** | **Strongly agree** |
| --- | --- | --- | --- | --- | --- |
| They can be used at any time, in any location | 1 | 2 | 3 | 4 | 5 |
| They can help me take control over my mental health | 1 | 2 | 3 | 4 | 5 |
| I can be more honest about how I am feeling | 1 | 2 | 3 | 4 | 5 |
| They can give me the opportunity to record and reflect on symptoms and experiences over time | 1 | 2 | 3 | 4 | 5 |
| They can give me the opportunity to share information in ‘real-time’ with my clinical/mental health team | 1 | 2 | 3 | 4 | 5 |
| They can give me the opportunity to identify triggers and patterns | 1 | 2 | 3 | 4 | 5 |
| My information is more private compared to talking to a member of my clinical/mental health team | 1 | 2 | 3 | 4 | 5 |
| Wearable devices are less stigmatising than attending therapy /seeing a member of my clinical/mental health team | 1 | 2 | 3 | 4 | 5 |

1. To what extent do you agree or disagree (1 = strongly disagree; 5 = strongly agree) that the following barriers would affect the likelihood of you using a wearable device? (Please tick all that apply)

| **Barrier** | **Strongly disagree**  **(Is not a barrier for me)** | **Disagree** | **Neutral** | **Agree** | **Strongly agree**  **(Is a barrier for me)** |
| --- | --- | --- | --- | --- | --- |
| Wearable device costs | 1 | 2 | 3 | 4 | 5 |
| Wearable device technology skills | 1 | 2 | 3 | 4 | 5 |
| Reading difficulties | 1 | 2 | 3 | 4 | 5 |
| Physical problems (eg, poor eyesight, hand tremors) | 1 | 2 | 3 | 4 | 5 |
| Lack of motivation | 1 | 2 | 3 | 4 | 5 |
| Forgetting to use the wearable device | 1 | 2 | 3 | 4 | 5 |
| Concerns about how helpful a wearable device would be | 1 | 2 | 3 | 4 | 5 |
| Concerns that a wearable device would be used as an excuse to replace face-to-face support | 1 | 2 | 3 | 4 | 5 |
| Concerns about where the information I put in the wearable device would go/who would get access | 1 | 2 | 3 | 4 | 5 |
| Concerns that the wearable device could be hacked | 1 | 2 | 3 | 4 | 5 |
| Feeling suspicious or paranoid about wearable devices in general | 1 | 2 | 3 | 4 | 5 |
| Feeling suspicious or paranoid about the wearable device specifically | 1 | 2 | 3 | 4 | 5 |
| Already able to manage mental health - do not need a wearable device | 1 | 2 | 3 | 4 | 5 |
| Concerns about focussing too much on symptoms | 1 | 2 | 3 | 4 | 5 |
| Concerns about being unable to personalise/tailor the wearable device | 1 | 2 | 3 | 4 | 5 |

1. Please list any reasons why you might want to use a wearable device to manage your mental health.

……………………………………………………………………………………………………………………………………………………………………………………………………………………………………………………………………………………………

1. Do you have ideas for any other ways that a wearable device could help you manage your mental health and/or improve your wellbeing?

………………………………………………………………………………………………………………………………………………………………………………………………………………………………………………………………………………………………

1. If a member of your clinical/mental health team asked you to use a smartphone app to help you manage your mental health between medical/health appointments (i.e. in your day-to-day life), would you wear/use it? (please tick to indicate)

Yes No (if no, please skip to Q33) Unsure

1. If you were offered a smartphone app as part of your mental health care, is there any extra mental health support you would want to receive? (please tick to indicate)
   - I would be happy to use the smartphone app on its own with no other mental health support
   - I would want to use the smartphone app in conjunction with remote mental health support (eg, therapy/Dr appointments over the phone / video-calling)
   - I would want to use the smartphone app in conjunction with face-to-face support/therapy/appointments
   - I would not want to use a smartphone app as part of my mental health care
2. To what extent do you agree or disagree with the following statements about smartphone apps for mental health care (1 = strongly disagree; 5 = strongly agree)?

| **Question** | **Strongly disagree** | **Disagree** | **Neutral** | **Agree** | **Strongly agree** |
| --- | --- | --- | --- | --- | --- |
| Using a smartphone app makes me depressed or anxious | 1 | 2 | 3 | 4 | 5 |
| Using a smartphone app makes me happy | 1 | 2 | 3 | 4 | 5 |
| Using a smartphone app makes me feel paranoid or suspicious | 1 | 2 | 3 | 4 | 5 |
| Using a smartphone app helps me to feel connected | 1 | 2 | 3 | 4 | 5 |
| Using a smartphone app helps my overall mental health and wellbeing | 1 | 2 | 3 | 4 | 5 |
| Using a smartphone app is unhelpful for my overall mental health and well-being | 1 | 2 | 3 | 4 | 5 |

1. To what extent do you agree or disagree (1 = strongly disagree; 5 = strongly agree) that the following are potential advantages of smartphone apps for mental healthcare? (Please tick all that apply)

|  | **Strongly disagree** | **Disagree** | **Neutral** | **Agree** | **Strongly agree** |
| --- | --- | --- | --- | --- | --- |
| Smartphone apps can be used at any time, in any location | 1 | 2 | 3 | 4 | 5 |
| Smartphone apps can help me take control of my mental health | 1 | 2 | 3 | 4 | 5 |
| I can be more honest in how I am feeling with a smartphone app | 1 | 2 | 3 | 4 | 5 |
| Smartphone apps can give me the opportunity to record and reflect on symptoms and experiences over time | 1 | 2 | 3 | 4 | 5 |
| Smartphone apps can give me the opportunity to share information in ‘real-time’ with my clinical/mental health team | 1 | 2 | 3 | 4 | 5 |
| Smartphone apps can give me the opportunity to identify triggers and patterns | 1 | 2 | 3 | 4 | 5 |
| Information is more private with a smartphone app compared to talking to a member of my clinical/mental health team | 1 | 2 | 3 | 4 | 5 |
| Smartphone apps are less stigmatising than attending therapy /seeing a member of my clinical/mental health team | 1 | 2 | 3 | 4 | 5 |

1. Smartphone apps can prompt/remind you to answer questions about your thoughts/feelings/behaviours/general health/whereabouts. Would you prefer to receive reminders to answer questions about your thoughts/feelings, etc from a smartphone app or would you prefer to answer these questions in your own time (ie, without being prompted/reminded)?

- I would like to receive prompts/reminders
- I would like to answer questions in my own time (ie, when I choose)
- I would like a combination
- I don’t mind
- Neither

1. To what extent do you agree or disagree (1 = strongly disagree; 5 = strongly agree) that the following are potential advantages of smartphone apps for mental health care?

| **Question** | **Strongly disagree** | **Disagree** | **Neutral** | **Agree** | **Strongly agree** |
| --- | --- | --- | --- | --- | --- |
| Ability to access a smartphone app at any time | 1 | 2 | 3 | 4 | 5 |
| Opportunity to take control over mental health | 1 | 2 | 3 | 4 | 5 |
| Opportunity to increase understanding about own symptoms and experiences | 1 | 2 | 3 | 4 | 5 |
| More comfortable providing honest responses to an anonymous/faceless device | 1 | 2 | 3 | 4 | 5 |
| Opportunity to record, and reflect back on, symptoms and experiences over time | 1 | 2 | 3 | 4 | 5 |
| Opportunity to take up-to-date records of symptoms and experiences to clinicians | 1 | 2 | 3 | 4 | 5 |
| Opportunity to identify triggers and patterns | 1 | 2 | 3 | 4 | 5 |
| Potential for staff to intervene early if increases in symptoms are identified | 1 | 2 | 3 | 4 | 5 |
| Increased privacy in comparison to paper-based symptom monitoring or therapy materials | 1 | 2 | 3 | 4 | 5 |

1. To what extent do you agree or disagree (1 = strongly disagree; 5 = strongly agree) that the following barriers would affect the likelihood of you using a mental health app? (Please tick all that apply)

| **Question** | **Strongly disagree** | **Disagree** | **Neutral** | **Agree** | **Strongly agree** |
| --- | --- | --- | --- | --- | --- |
| Smartphone handset costs | 1 | 2 | 3 | 4 | 5 |
| Smartphone data costs | 1 | 2 | 3 | 4 | 5 |
| Poor storage for apps on smartphone | 1 | 2 | 3 | 4 | 5 |
| Smartphone technology skills | 1 | 2 | 3 | 4 | 5 |
| Reading difficulties | 1 | 2 | 3 | 4 | 5 |
| Physical problems (eg, poor eyesight, hand tremors) | 1 | 2 | 3 | 4 | 5 |
| Lack of motivation | 1 | 2 | 3 | 4 | 5 |
| Forgetting to use the app | 1 | 2 | 3 | 4 | 5 |
| Concerns about how helpful a mental health app would be | 1 | 2 | 3 | 4 | 5 |
| Concerns that a mental health app would be used as an excuse to replace face-to-face support | 1 | 2 | 3 | 4 | 5 |
| Concerns about where the information I put in the app would go/who would get access | 1 | 2 | 3 | 4 | 5 |
| Concerns that the app could be hacked | 1 | 2 | 3 | 4 | 5 |
| Feeling suspicious or paranoid about mobile phones in general | 1 | 2 | 3 | 4 | 5 |
| Feeling suspicious or paranoid about the app specifically | 1 | 2 | 3 | 4 | 5 |
| Already able to manage mental health - do not need a mental health app | 1 | 2 | 3 | 4 | 5 |
| Concerns about focusing too much on symptoms | 1 | 2 | 3 | 4 | 5 |
| Concerns about being unable to personalise/tailor mental health apps | 1 | 2 | 3 | 4 | 5 |

1. Please list any reasons why you might want to use a smartphone app to manage your mental health.

………………………………………………………………………………………………………………………………………………………………………………………………………………………………………………………………………………………………

1. Do you have any ideas for any other ways that a smartphone app could help you manage your mental health?

………………………………………………………………………………………………………………………………………………………………………………………………………………………………………………………………………………………………

# Part 4: Type of Wearable

Some people use wearable devices or smartwatches for things like counting steps, helping with their sleep routine, setting medication reminders, or ordering prescriptions. There are also special devices that people can wear to track things like their blood sugar levels or heart rhythm.

Here are some examples of what wearable devices look like:


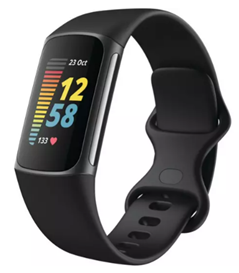

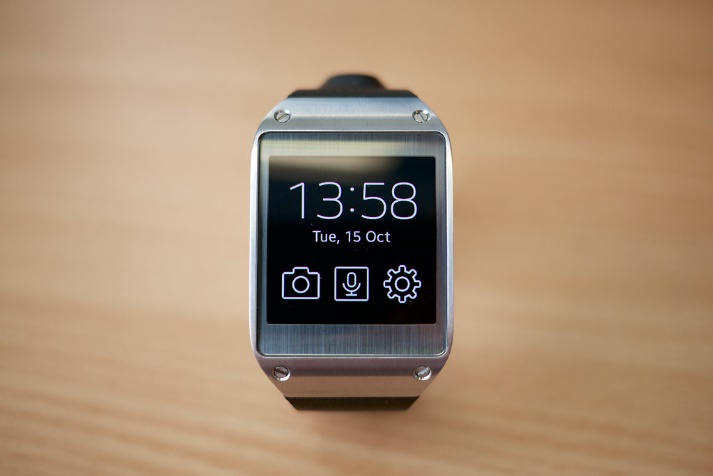


"Samsung Galaxy Gear smartwatch" by Janitors is marked with CC BY 2.0.

“Apple Watch" by wiyre.com is licensed under CC BY 2.0

1. Look at the pictures below. If you were to use a wearable device, which of these appeals to you the most?

| Empatica E4  “[Empatica E4](https://www.flickr.com/photos/99658898@N00/28348172315)" by [osiristhe](https://www.flickr.com/photos/99658898@N00) is marked with [CC BY-ND 2.0](https://creativecommons.org/licenses/by-nd/2.0/?ref=openverse).  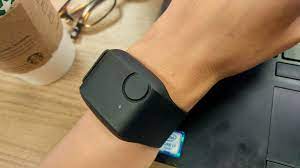  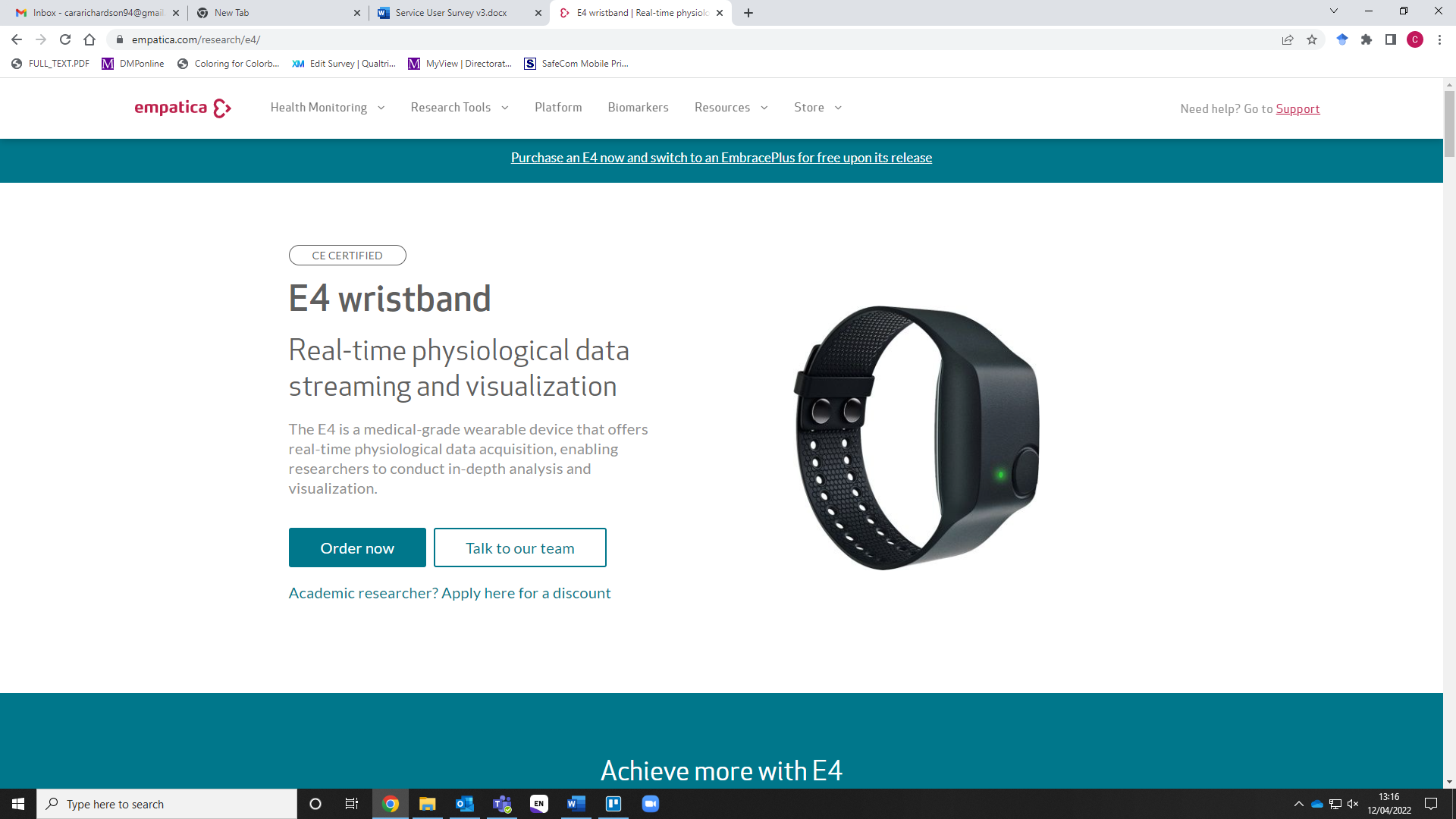 | Fitbit  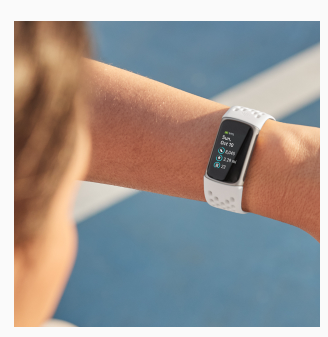  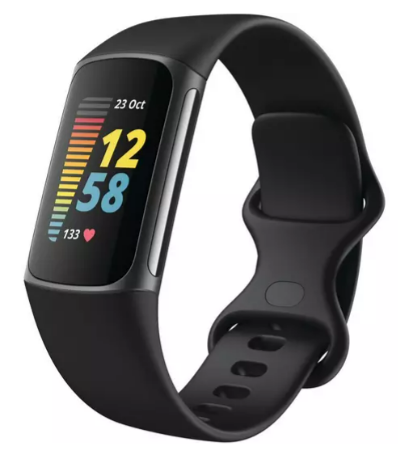 |  |
| --- | --- | --- |
| - I would not wear or use a wearable device (If ticked, skip to Q37). | |  |
| [If ticked yes for the Empatica E4 device] Please complete the following questions about the Empatica E4 device: | | |
| *How often do you think you would wear/use it?*   - Every day - A few days a week - Once a week - Once a fortnight - Once a month - Other. Please state ……………………………. | | |
| *How long do you think you would wear/use it for?*   - A week - A month - 3 months - 6 months - 1 year - Longer - Other Please state…………………………. | | |
| [If ticked yes for the Fitbit device] Please complete the following questions about the Fitbit device: | | |
| *How often do you think you would wear/ use it?*   - Every day - A few days a week - Once a week - Once a fortnight - Once a month - Other ……………………………. | | |
| *How long do you think you would wear/use it for?*   - A week - A month - 3 months - 6 months - 1 year - Longer - Other. Please state………………… | | |

1. Out of the 3 Fitbit devices below, which colour do you prefer?

| 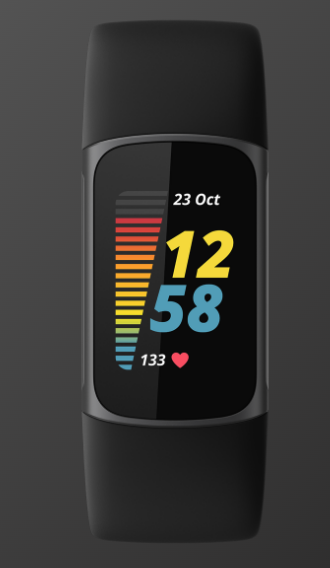   - Black/Graphite Stainless Steel | 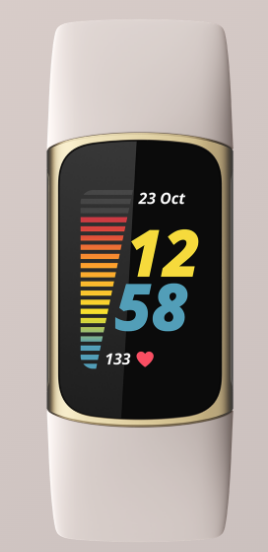   - Lunar White/Soft Gold Stainless Steel | 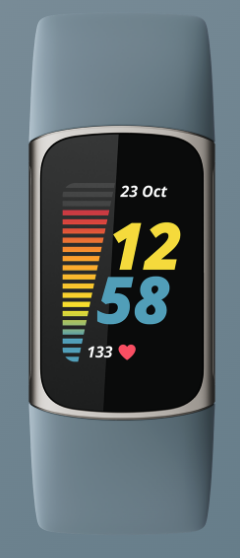   - Steel Blue/Platinum Stainless Steel |
| --- | --- | --- |

1. If someone gave you the choice of a wearable device to use, what features would be important to you? Please tick the following from 1 (not important) to 5 (very important).

***For a wearable device, I would like one which:***

| **Question** | **N/A** | **Not Important** | **Somewhat important** | **Not bothered** | **Important** | **Very Important** | **Do you have any concerns/issues with the collection of this type of information?** |
| --- | --- | --- | --- | --- | --- | --- | --- |
| has a long battery life (works for a long time before it needs to be charged again) | 0 | 1 | 2 | 3 | 4 | 5 | ………………………………… |
| is easy to use | 0 | 1 | 2 | 3 | 4 | 5 | ………………………………… |
| keeps my data/information private | 0 | 1 | 2 | 3 | 4 | 5 | ………………………………… |
| I feel confident using | 0 | 1 | 2 | 3 | 4 | 5 | ………………………………… |
| is similar to one my friends/family/peers use | 0 | 1 | 2 | 3 | 4 | 5 | ………………………………… |
| doesn’t take long to charge | 0 | 1 | 2 | 3 | 4 | 5 | ………………………………… |
| has a certain look (e.g. the size and/or shape of the device) | 0 | 1 | 2 | 3 | 4 | 5 | ………………………………… |
| nudges/reminds me to complete a certain task (e.g. exercise or take medication) | 0 | 1 | 2 | 3 | 4 | 5 | ………………………………… |
| connects to the internet | 0 | 1 | 2 | 3 | 4 | 5 | ………………………………… |
| has customisation/personalisation options e.g. change the size of the text, layout/colours of the watch face | 0 | 1 | 2 | 3 | 4 | 5 | ………………………………… |
| was a reasonable price | 0 | 1 | 2 | 3 | 4 | 5 | ………………………………… |
| can measure health information (e.g. heart rate) | 0 | 1 | 2 | 3 | 4 | 5 | ………………………………… |
| allows me to use my credit or debit card from the device (e.g. apple pay, fitbit pay) | 0 | 1 | 2 | 3 | 4 | 5 | ………………………………… |
| connects to my smartphone | 0 | 1 | 2 | 3 | 4 | 5 | ………………………………… |
| tracks my sleep | 0 | 1 | 2 | 3 | 4 | 5 | ………………………………… |
| tracks my steps | 0 | 1 | 2 | 3 | 4 | 5 | ………………………………… |
| has a clock (alarm and timer) | 0 | 1 | 2 | 3 | 4 | 5 | ………………………………… |
| allows me to read text messages or take phone calls | 0 | 1 | 2 | 3 | 4 | 5 | ………………………………… |
| is water resistant | 0 | 1 | 2 | 3 | 4 | 5 | ………………………………… |
| can stream music (i.e connect to Spotify or Apple music) | 0 | 1 | 2 | 3 | 4 | 5 | ………………………………… |
| measures heart rate (pulse) | 0 | 1 | 2 | 3 | 4 | 5 | ………………………………… |
| logs/tracks exercise | 0 | 1 | 2 | 3 | 4 | 5 | ………………………………… |
| logs mood score | 0 | 1 | 2 | 3 | 4 | 5 | ………………………………… |
| tracks menstrual cycle | 0 | 1 | 2 | 3 | 4 | 5 | ………………………………… |
| measures blood oxygen level (indicates the amount of oxygen that your blood cells are carrying around your body) | 0 | 1 | 2 | 3 | 4 | 5 | ………………………………… |
| tracks my location (GPS sensor) | 0 | 1 | 2 | 3 | 4 | 5 | ………………………………… |
| tracks breathing rate | 0 | 1 | 2 | 3 | 4 | 5 | ………………………………… |
| tracks how much you sweat (perspiration) e.g. to help understand how your body responds to physical activity) | 0 | 1 | 2 | 3 | 4 | 5 | ………………………………… |
| tracks temperature | 0 | 1 | 2 | 3 | 4 | 5 | ………………………………… |
| is comfortable to wear (e.g. the material of the device is comfortable) | 0 | 1 | 2 | 3 | 4 | 5 | ………………………………… |
| measures blood volume (Photoplethysmography sensor) | 0 | 1 | 2 | 3 | 4 | 5 | ………………………………… |

1. Are there any other features of wearable devices that are important to you?

**……………………………………………………………………………………………………………………………………………………………………………………………………………………………………………………………………………………**

# Part 5: How Digital Health Tools/Platforms Collect and Use Data

In this section, we will ask you about the ways University researchers collect and analyse the data collected by digital health tools/platforms (eg, smartphone apps or smartwatches).

1. Remote Symptom Monitoring

One way smartphones can be used is to keep track of how you have been feeling day-to-day. Remote symptom monitoring is a healthcare delivery method that uses technology to monitor people’s thoughts, feelings, behaviours, or health in general outside of a traditional clinical setting. How this works is that an app prompts/sends a notification to you each day to answer questions about your mood and feelings (eg, whether you’ve been feeling low or bothered by voices). It can also ask you about where you are and who you are with (eg, family members, friends). By responding to prompts/notifications, the app keeps track over time of how you are feeling or what you have been thinking at any time or location. It is possible to then share this information securely with a member of your clinical/mental health team.

The following questions will ask how you feel about ‘remote symptom monitoring’ and sharing information collected in this way with a member of your clinical/mental health team or a University researcher.

1. How comfortable do you feel with University researchers (who are conducting a study you agreed to be a part of) using a ‘remote symptom monitoring’ app to collect information about your feelings/behaviours/general health/whereabouts? (please tick to indicate)
2. Comfortable Uncomfortable Unsure
3. How helpful would a ‘remote symptom monitoring’ app be in helping you manage your mental health/well-being? (please tick to indicate)

Helpful Unhelpful Unsure

1. Would you be comfortable with information about your thoughts/feelings/behaviours/general health/whereabouts being collected via… (please tick all that apply):

Smartphone Wearable device, e.g., Fitbit or Apple Watch

Both Neither

1. How comfortable do you feel with information about your thoughts/feelings/behaviours/general health/whereabouts collected via a ‘remote symptoms monitoring’ app being shared with a member of your clinical/mental health team? (please tick to indicate):

Comfortable Uncomfortable Unsure

1. If you have any comments or concerns about this, please describe these in the space below.

…………………………………………………………………………………..

1. Passive Sensing

Smartphones and wearables (like Fitbits or smartwatches) can gather lots of round-the-clock (continuous) information, without you having to *do* anything apart from wear the wearable or carry your phone around with you. This is called passive sensing.

This information tells a story about the kind of things you have been doing – like whether you’ve been sleeping, exercising, travelling, or phoning people – and can give clues about how you might be feeling. For example, someone who is feeling very anxious or low might not feel like going out much or seeing people. They might stay at home more than usual and phone their friends less.

Because their smartphone can gather information about where they have been and how many phone calls they’ve made, it could quickly spot that change. Allowing their phone to spot changes like this could help the person (and their mental health team) know that they might need some extra support.

1. How comfortable do you feel with University researchers (who are conducting a study you agreed to be a part of) using passive sensing to collect information about your feelings/behaviours/general health/whereabouts? (please tick to indicate)
2. Comfortable Uncomfortable Unsure
3. How helpful would passive sensing be in helping you manage your mental health/well-being? (please tick to indicate)

Helpful Unhelpful Unsure

1. Would you be comfortable with information about whether you’ve been sleeping, exercising, travelling, or phoning people being collected via… (please tick all that apply):

Smartphone Wearable device e.g. Fitbit or Apple watch

Both Neither

1. How comfortable do you feel with information about whether you’ve been sleeping, exercising, travelling, or phoning people collected via passive sensing being shared with a member of your clinical/mental health team? (please tick to indicate):

Comfortable Uncomfortable Unsure

1. If you have any comments or concerns about this, please describe these in the space below.

…………………………………………………………………………………..

1. To what extent do you agree with the following statements?

*I am comfortable with University researchers (who are conducting a study you agreed to be a part of) using a smartphone or wearable device to collect information about me related to my experiences of (please tick your response):*

| **Question** | **Strongly disagree** | **Disagree** | **Neutral** | **Agree** | **Strongly agree** | **Do you have any concerns/issues with the collection of this type of information?** |
| --- | --- | --- | --- | --- | --- | --- |
| hearing voices (or other sensory experiences like visions, etc) | 1 | 2 | 3 | 4 | 5 | …………………………. |
| having unusual beliefs | 1 | 2 | 3 | 4 | 5 | …………………………. |
| feeling suspicious or distrustful of others | 1 | 2 | 3 | 4 | 5 | …………………………. |
| my mood | 1 | 2 | 3 | 4 | 5 | …………………………. |
| Fears I might have about my mental health deteriorating | 1 | 2 | 3 | 4 | 5 | …………………………. |
| who I am with (e.g. how often I meet with friends and family) | 1 | 2 | 3 | 4 | 5 | …………………………. |
| my activity levels (e.g. floors climbed, step count, minutes active or minutes sitting down) | 1 | 2 | 3 | 4 | 5 | …………………………. |
| my heart rate | 1 | 2 | 3 | 4 | 5 | …………………………. |
| my sleep patterns | 1 | 2 | 3 | 4 | 5 | …………………………. |
| my approximate location (e.g. at home, 1 mile from home) | 1 | 2 | 3 | 4 | 5 | …………………………. |
| any significant location changes (e.g. a place you haven’t been to before) | 1 | 2 | 3 | 4 | 5 | …………………………. |
| the weather where I am (e.g. air temperature, air pressure, humidity) | 1 | 2 | 3 | 4 | 5 | …………………………. |
| how bright it is where I am | 1 | 2 | 3 | 4 | 5 | …………………………. |
| how often i use my phone (e.g. how often you use your mobile phone apps) | 1 | 2 | 3 | 4 | 5 | …………………………. |
| which apps I use | 1 | 2 | 3 | 4 | 5 | …………………………. |
| how often I make phone calls | 1 | 2 | 3 | 4 | 5 | …………………………. |
| how often I send messages (either sms or on what’s app, snapchat or any other message app) | 1 | 2 | 3 | 4 | 5 | …………………………. |
| my contacts list | 1 | 2 | 3 | 4 | 5 | …………………………. |
| how many Bluetooth devices my phone detects each day (e.g. Bluetooth can potentially give an indication of how many other people you are near to in the day) | 1 | 2 | 3 | 4 | 5 | …………………………. |
| my phone or wearable device battery level | 1 | 2 | 3 | 4 | 5 | …………………………. |

# Part 6: Questions about you

| 1. **Which country do you live in?**  - United Kingdom - USA - Australia - Europe - Other. Please state……………………….. - Prefer not to say | 1. **How did you hear about this survey?**  - Through a GP (General Practitioner) or GP Surgery - Through a Mental Health NHS Trust or Mental Health Team - Through a charity (eg, MIND) - Social media - Through another way |
| --- | --- |
| 1. **Please write the name of the city or town you live in:**   ……………………………………………………….. | 1. **Please write the name of your GP’s Practice here (e.g. Lakelodge Medical Practice):**   ……………………………………………………….. |
| 1. **Please write the name of the NHS Trust or Health Board here (e.g. Greater Manchester Mental Health):**   ……………………………………………………….. |  |

### **Figure S1. Principal component analysis of participant comfort with sharing data: PC 1 and PC 2.**

**
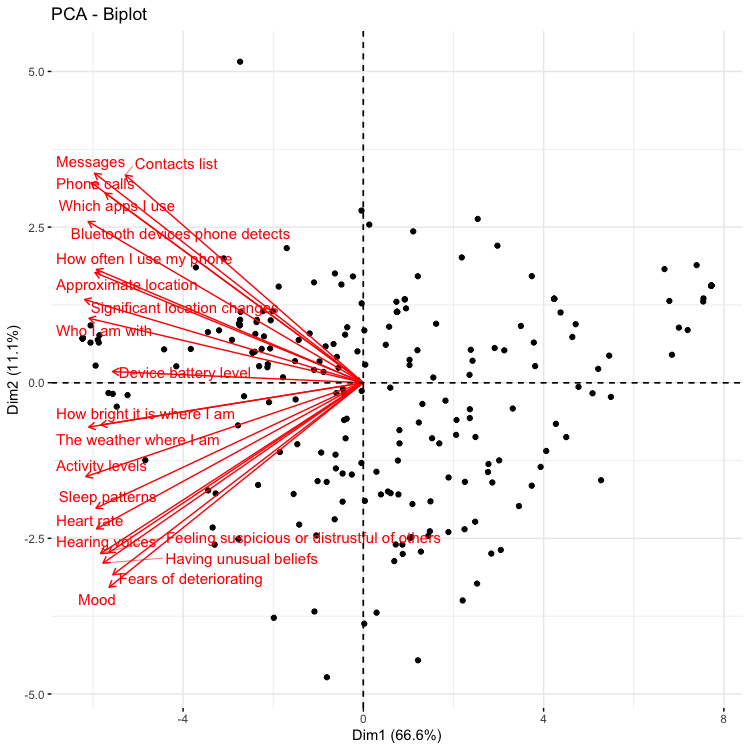
**

### **Figure S2. Principal component analysis of participant comfort with sharing data: PC 2 and PC 3.**

**
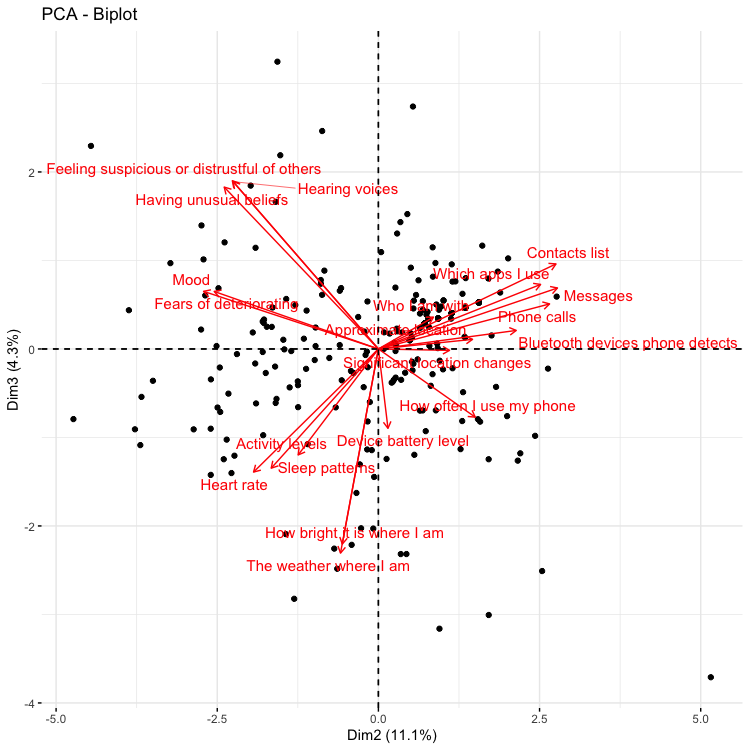
**

### **Table S2. Concerns about collecting data using active monitoring (total number of references=91).**

| **Coding** | **Number of coding references, n (%)** |
| --- | --- |
| Risk of data misuse | 29 (32) |
| Feeling uncomfortable with being monitored | 19 (21) |
| Tracking symptom making people feel worse | 14 (15) |
| Lack of trust with data recipients | 12 (13) |
| Risk of replacing face-to-face | 12 (13) |
| Unsure about the value of digital monitoring | 4 (4) |
| NHS lacks the capacity to support digital monitoring | 1 (1) |

### **Table S3. Concerns about collecting data using passive sensing methods** **(total number of references=57).**

| **Coding** | **Number of coding references, n (%)** |
| --- | --- |
| Feeling uncomfortable with passive sensing | 19 (33) |
| Invasive and unhelpful | 13 (23) |
| Preferring to use in a collaborative way | 9 (16) |
| Worried about data misuse | 7 (12) |
| Unsure about the value of passive sensing | 4 (7) |
| Risk of replacing face-to-face | 3 (5) |
| Increase inequity | 1 (2) |
| Information overload | 1 (2) |

### **Table S4. Potential advantages of smartphone apps/wearable devices for mental healthcare: N (%).**

| **Items** | **Smartphone app (n = 240)** | | | | | | **Wearable device (n = 248)** | | | | | |
| --- | --- | --- | --- | --- | --- | --- | --- | --- | --- | --- | --- | --- |
|  | **Strongly disagree** | **Disagree** | **Neutral** | **Agree** | **Strongly agree** | **Missing** | **Strongly disagree** | **Disagree** | **Neutral** | **Agree** | **Strongly agree** | **Missing** |
| They can be used at any time, in any location | 5 (2.08) | 10 (4.17) | 33 (13.75) | 127 (52.92) | 62 (25.83) | 3 (1.25) | 4 (1.61) | 10 (4.03) | 34 (13.71) | 124 (50) | 69 (27.82) | 7 (2.82) |
| They can help me take control over my mental health | 7 (2.92) | 26 (10.83) | 81 (33.75) | 87 (36.25) | 35 (14.58) | 4 (1.67) | 10 (4.03) | 22 (8.87) | 87 (35.08) | 84 (33.87) | 37 (14.92) | 8 (3.23) |
| I can be more honest in how I am feeling | 8 (3.33) | 37 (15.42) | 78 (32.5) | 84 (35) | 29 (12.08) | 4 (1.67) | 8 (3.23) | 26 (10.48) | 74 (29.84) | 100 (40.32) | 32 (12.9) | 8 (3.23) |
| They can give me the opportunity to record and reflect on symptoms and experiences over time | 2 (0.83) | 10 (4.17) | 48 (20) | 117 (48.75) | 59 (24.58) | 4 (1.67) | 6 (2.42) | 21 (8.47) | 44 (17.74) | 112 (45.16) | 56 (22.58) | 9 (3.63) |
| They can give me the opportunity to share information in ‘real-time’ with my clinical/mental health team | 3 (1.25) | 8 (3.33) | 45 (18.75) | 134 (55.83) | 46 (19.17) | 4 (1.67) | 7 (2.82) | 16 (6.45) | 37 (14.92) | 127 (51.21) | 53 (21.37) | 8 (3.23) |
| They can give me the opportunity to identify triggers and patterns | 4 (1.67) | 7 (2.92) | 54 (22.5) | 119 (49.58) | 52 (21.67) | 4 (1.67) | 3 (1.21) | 13 (5.24) | 52 (20.97) | 107 (43.15) | 65 (26.21) | 8 (3.23) |
| My information is more private compared to talking to a member of my clinical/mental health team | 25 (10.42) | 66 (27.5) | 78 (32.5) | 48 (20) | 19 (7.92) | 4 (1.67) | 22 (8.87) | 45 (18.15) | 94 (37.9) | 48 (19.35) | 29 (11.69) | 10 (4.03) |
| They are less stigmatising than attending therapy /seeing a member of my clinical/mental health team | 21 (8.75) | 58 (24.17) | 72 (30) | 67 (27.92) | 18 (7.5) | 4 (1.67) | 24 (9.68) | 59 (23.79) | 70 (28.23) | 63 (25.4) | 23 (9.27) | 9 (3.63) |

### **Table S5. Additional advantages specific to smartphone apps (n=240): N (%).**

| **Items** | **Strongly disagree** | **Disagree** | **Neutral** | **Agree** | **Strongly agree** | **Missing** |
| --- | --- | --- | --- | --- | --- | --- |
| Ability to access a smartphone app at any time | 7 (2.92) | 3 (1.25) | 42 (17.5) | 115 (47.92) | 66 (27.5) | 7 (2.92) |
| Opportunity to take control over mental health | 3 (1.25) | 17 (7.08) | 78 (32.5) | 95 (39.58) | 39 (16.25) | 8 (3.33) |
| Opportunity to increase understanding about own symptoms and experiences | 3 (1.25) | 11 (4.58) | 53 (22.08) | 114 (47.5) | 51 (21.25) | 8 (3.33) |
| More comfortable providing honest responses to an anonymous/faceless device | 14 (5.83) | 28 (11.67) | 79 (32.92) | 79 (32.92) | 32 (13.33) | 8 (3.33) |
| Opportunity to record, and reflect back on, symptoms and experiences over time | 3 (1.25) | 15 (6.25) | 40 (16.67) | 110 (45.83) | 64 (26.67) | 8 (3.33) |
| Opportunity to take up-to-date records of symptoms and experiences to clinicians | 3 (1.25) | 6 (2.5) | 48 (20) | 116 (48.33) | 59 (24.58) | 8 (3.33) |
| Opportunity to identify triggers and patterns | 3 (1.25) | 8 (3.33) | 40 (16.67) | 121 (50.42) | 60 (25) | 8 (3.33) |
| Potential for staff to intervene early if increases in symptoms are identified | 5 (2.08) | 13 (5.42) | 37 (15.42) | 117 (48.75) | 60 (25) | 8 (3.33) |
| Increased privacy in comparison to paper-based symptom monitoring or therapy materials | 16 (6.67) | 29 (12.08) | 77 (32.08) | 76 (31.67) | 34 (14.17) | 8 (3.33) |

### **Table S6. Barriers to using smartphone apps/wearable devices for mental healthcare: N (%).**

| **Items** | **Smartphone app (n = 240)** | | | | | | **Wearable device (n = 248)** | | | | | |
| --- | --- | --- | --- | --- | --- | --- | --- | --- | --- | --- | --- | --- |
|  | **Strongly disagree** | **Disagree** | **Neutral** | **Agree** | **Strongly agree** | **Missing** | **Strongly disagree** | **Disagree** | **Neutral** | **Agree** | **Strongly agree** | **Missing** |
| Costs | 35 (14.58) | 39 (16.25) | 50 (20.83) | 76 (31.67) | 29 (12.08) | 11 (4.58) | 26 (10.48) | 22 (8.87) | 46 (18.55) | 79 (31.85) | 65 (26.21) | 10 (4.03) |
| Technology skills | 53 (22.08) | 50 (20.83) | 58 (24.17) | 52 (21.67) | 15 (6.25) | 12 (5) | 60 (24.19) | 42 (16.94) | 61 (24.6) | 53 (21.37) | 20 (8.06) | 12 (4.84) |
| Reading difficulties | 82 (34.17) | 70 (29.17) | 40 (16.67) | 25 (10.42) | 11 (4.58) | 12 (5) | 105 (42.34) | 55 (22.18) | 41 (16.53) | 25 (10.08) | 10 (4.03) | 12 (4.84) |
| Physical problems (e.g. poor eyesight, hand tremors) | 80 (33.33) | 62 (25.83) | 46 (19.17) | 29 (12.08) | 11 (4.58) | 12 (5) | 103 (41.53) | 54 (21.77) | 39 (15.73) | 30 (12.1) | 10 (4.03) | 12 (4.84) |
| Lack of motivation | 15 (6.25) | 38 (15.83) | 65 (27.08) | 88 (36.67) | 22 (9.17) | 12 (5) | 39 (15.73) | 38 (15.32) | 56 (22.58) | 75 (30.24) | 29 (11.69) | 11 (4.44) |
| Forgetting to use the wearable device | 13 (5.42) | 39 (16.25) | 46 (19.17) | 104 (43.33) | 26 (10.83) | 12 (5) | 36 (14.52) | 38 (15.32) | 57 (22.98) | 81 (32.66) | 25 (10.08) | 11 (4.44) |
| Concerns about how helpful it would be | 18 (7.5) | 38 (15.83) | 69 (28.75) | 81 (33.75) | 22 (9.17) | 12 (5) | 40 (16.13) | 42 (16.94) | 58 (23.39) | 79 (31.85) | 17 (6.85) | 12 (4.84) |
| Concerns that it would be used as an excuse to replace face-to-face support | 16 (6.67) | 32 (13.33) | 39 (16.25) | 92 (38.33) | 49 (20.42) | 12 (5) | 27 (10.89) | 29 (11.69) | 59 (23.79) | 77 (31.05) | 44 (17.74) | 12 (4.84) |
| Concerns about the where the information I put in it would go/who would get access | 19 (7.92) | 31 (12.92) | 40 (16.67) | 95 (39.58) | 43 (17.92) | 12 (5) | 28 (11.29) | 29 (11.69) | 46 (18.55) | 87 (35.08) | 47 (18.95) | 11 (4.44) |
| Concerns that it could be hacked | 17 (7.08) | 43 (17.92) | 42 (17.5) | 82 (34.17) | 44 (18.33) | 12 (5) | 34 (13.71) | 41 (16.53) | 39 (15.73) | 83 (33.47) | 39 (15.73) | 12 (4.84) |
| Feeling suspicious or paranoid about the technology in general | 45 (18.75) | 58 (24.17) | 53 (22.08) | 51 (21.25) | 21 (8.75) | 12 (5) | 53 (21.37) | 59 (23.79) | 48 (19.35) | 55 (22.18) | 21 (8.47) | 12 (4.84) |
| Feeling suspicious or paranoid about the technology specifically | 38 (15.83) | 57 (23.75) | 59 (24.58) | 50 (20.83) | 24 (10) | 12 (5) | 57 (22.98) | 59 (23.79) | 56 (22.58) | 44 (17.74) | 20 (8.06) | 12 (4.84) |
| Already able to manage mental health - do not need one | 30 (12.5) | 56 (23.33) | 82 (34.17) | 43 (17.92) | 17 (7.08) | 12 (5) | 34 (13.71) | 47 (18.95) | 74 (29.84) | 62 (25) | 20 (8.06) | 11 (4.44) |
| Concerns about focusing too much on symptoms | 14 (5.83) | 53 (22.08) | 74 (30.83) | 70 (29.17) | 17 (7.08) | 12 (5) | 28 (11.29) | 38 (15.32) | 77 (31.05) | 74 (29.84) | 19 (7.66) | 12 (4.84) |
| Concerns about being unable to personalise/tailor the wearable device | 23 (9.58) | 42 (17.5) | 73 (30.42) | 69 (28.75) | 21 (8.75) | 12 (5) | 45 (18.15) | 45 (18.15) | 66 (26.61) | 63 (25.4) | 18 (7.26) | 11 (4.44) |
| Smartphone data costs | 29 (12.08) | 42 (17.5) | 56 (23.33) | 74 (30.83) | 27 (11.25) | 12 (5) | – | – | – | – | – | – |
| Poor storage for apps on smartphone | 30 (12.5) | 54 (22.5) | 70 (29.17) | 51 (21.25) | 22 (9.17) | 13 (5.42) | – | – | – | – | – | – |

### **Table S7. Perceived importance of certain features of wearable devices (n=209): N (%).**

| **Items** | **Not applicable** | **Not Important** | **Somewhat Important** | **Not Bothered** | **Important** | **Very Important** | **Missing** |
| --- | --- | --- | --- | --- | --- | --- | --- |
| Keeps my data/information private | - | 2 (0.96) | 8 (3.83) | 11 (5.26) | 58 (27.75) | 128 (61.24) | 2 (0.96) |
| Has a long battery life (works for a long time before it needs to be charged again) | 2 (0.96) | 5 (2.39) | 11 (5.26) | 8 (3.83) | 72 (34.45) | 109 (52.15) | 2 (0.96) |
| Is comfortable to wear (e.g. The material of the device is comfortable) | - | 2 (0.96) | 9 (4.31) | 11 (5.26) | 86 (41.15) | 99 (47.37) | 2 (0.96) |
| Is easy to use | 2 (0.96) | 6 (2.87) | 8 (3.83) | 17 (8.13) | 78 (37.32) | 96 (45.93) | 2 (0.96) |
| I feel confident using | 2 (0.96) | 3 (1.44) | 10 (4.78) | 12 (5.74) | 86 (41.15) | 94 (44.98) | 2 (0.96) |
| Was a reasonable price | 1 (0.48) | 3 (1.44) | 10 (4.78) | 12 (5.74) | 87 (41.63) | 94 (44.98) | 2 (0.96) |
| Can measure health information (e.g. Heart rate) | - | 6 (2.87) | 12 (5.74) | 16 (7.66) | 85 (40.67) | 87 (41.63) | 3 (1.44) |
| Has a clock (alarm and timer) | - | 6 (2.87) | 11 (5.26) | 24 (11.48) | 84 (40.19) | 82 (39.23) | 2 (0.96) |
| Is water resistant | 1 (0.48) | 8 (3.83) | 17 (8.13) | 21 (10.05) | 79 (37.8) | 81 (38.76) | 2 (0.96) |
| Tracks my steps | 1 (0.48) | 9 (4.31) | 11 (5.26) | 27 (12.92) | 79 (37.8) | 80 (38.28) | 2 (0.96) |
| Logs/tracks exercise | 1 (0.48) | 9 (4.31) | 14 (6.7) | 25 (11.96) | 80 (38.28) | 78 (37.32) | 2 (0.96) |
| Logs mood score | 3 (1.44) | 10 (4.78) | 16 (7.66) | 14 (6.7) | 88 (42.11) | 76 (36.36) | 2 (0.96) |
| Connects to the internet | 1 (0.48) | 19 (9.09) | 15 (7.18) | 29 (13.88) | 69 (33.01) | 74 (35.41) | 2 (0.96) |
| Doesn’t take long to charge | 4 (1.91) | 6 (2.87) | 27 (12.92) | 26 (12.44) | 73 (34.93) | 71 (33.97) | 2 (0.96) |
| Connects to my smartphone | 6 (2.87) | 18 (8.61) | 21 (10.05) | 20 (9.57) | 71 (33.97) | 71 (33.97) | 2 (0.96) |
| Measures heart rate (pulse) | 1 (0.48) | 9 (4.31) | 10 (4.78) | 22 (10.53) | 94 (44.98) | 71 (33.97) | 2 (0.96) |
| Tracks my sleep | - | 15 (7.18) | 18 (8.61) | 29 (13.88) | 77 (36.84) | 68 (32.54) | 2 (0.96) |
| Nudges/reminds me to complete a certain task (e.g. Exercise or take medication) | - | 7 (3.35) | 19 (9.09) | 21 (10.05) | 96 (45.93) | 64 (30.62) | 2 (0.96) |
| Measures blood oxygen level (indicates the amount of oxygen that your blood cells are carrying around your body) | 4 (1.91) | 21 (10.05) | 16 (7.66) | 27 (12.92) | 78 (37.32) | 61 (29.19) | 2 (0.96) |
| Tracks breathing rate | 1 (0.48) | 19 (9.09) | 16 (7.66) | 32 (15.31) | 82 (39.23) | 56 (26.79) | 3 (1.44) |
| Has customisation/personalisation options e.g. Change the size of the text, layout/colours of the watch face | 3 (1.44) | 22 (10.53) | 21 (10.05) | 35 (16.75) | 75 (35.89) | 51 (24.4) | 2 (0.96) |
| Tracks temperature | 3 (1.44) | 36 (17.22) | 16 (7.66) | 38 (18.18) | 67 (32.06) | 47 (22.49) | 2 (0.96) |
| Allows me to read text messages or take phone calls | 1 (0.48) | 31 (14.83) | 16 (7.66) | 41 (19.62) | 71 (33.97) | 46 (22.01) | 3 (1.44) |
| Tracks how much you sweat (perspiration) e.g. To help understand how your body responds to physical activity) | 4 (1.91) | 45 (21.53) | 12 (5.74) | 40 (19.14) | 63 (30.14) | 43 (20.57) | 2 (0.96) |
| Measures blood volume (Photoplethysmography sensor) | 5 (2.39) | 35 (16.75) | 11 (5.26) | 52 (24.88) | 61 (29.19) | 43 (20.57) | 2 (0.96) |
| Has a certain look (e.g. The size and/or shape of the device) | 9 (4.31) | 23 (11) | 22 (10.53) | 34 (16.27) | 78 (37.32) | 39 (18.66) | 4 (1.91) |
| Can stream music (i.e connect to Spotify or Apple music) | 5 (2.39) | 39 (18.66) | 13 (6.22) | 59 (28.23) | 52 (24.88) | 39 (18.66) | 2 (0.96) |
| Tracks my location (GPS sensor) | 7 (3.35) | 47 (22.49) | 19 (9.09) | 42 (20.1) | 55 (26.32) | 36 (17.22) | 3 (1.44) |
| Is similar to one my friends/family/peers use | 14 (6.7) | 57 (27.27) | 13 (6.22) | 64 (30.62) | 29 (13.88) | 30 (14.35) | 2 (0.96) |
| Tracks menstrual cycle | 74 (35.41) | 19 (9.09) | 11 (5.26) | 37 (17.7) | 36 (17.22) | 30 (14.35) | 2 (0.96) |
| Allows me to use my credit or debit card from the device (e.g. Apple Pay, Fitbit Pay) | 9 (4.31) | 65 (31.1) | 15 (7.18) | 47 (22.49) | 45 (21.53) | 26 (12.44) | 2 (0.96) |
